# Genome-wide evaluation of gene editing outcomes using CRISPR/Cas9 in seed propagated *Camelina sativa* and vegetatively propagated *Solanum tuberosum*

**DOI:** 10.3389/fpls.2024.1496861

**Published:** 2024-11-26

**Authors:** Thilani B. Jayakody, Daniel Zarka, Keun Ho Cho, Jacob Jensen, Samantha Sikora, C. Robin Buell, David S. Douches, Satya Swathi Nadakuduti

**Affiliations:** ^1^ Department of Plant, Soil and Microbial Sciences, Michigan State University, East Lansing, MI, United States; ^2^ Environmental Horticulture Department, University of Florida, Gainesville, FL, United States; ^3^ Center for Applied Genetic Technologies, University of Georgia, Athens, GA, United States; ^4^ Department of Crop & Soil Sciences, University of Georgia, Athens, GA, United States; ^5^ Institute of Plant Breeding, Genetics & Genomics, University of Georgia, Athens, GA, United States; ^6^ The Plant Center, University of Georgia, Athens, GA, United States; ^7^ Plant Molecular and Cellular Biology Program, University of Florida, Gainesville, FL, United States; ^8^ University of Florida Genetics Institute, Gainesville, FL, United States

**Keywords:** CRISPR/Cas9, gene-editing, off-target, *Agrobacterium*-mediated transformation, transgenerational editing, mosaic edits, somatic mutations

## Abstract

CRISPR/Cas9 is the most popular genome editing platform for investigating gene function or improving traits in plants. The specificity of gene editing has yet to be evaluated at a genome-wide scale in seed-propagated *Camelina sativa* (L.) Crantz (camelina) or clonally propagated *Solanum tuberosum* L. (potato). In this study, seven potato and nine camelina stable transgenic Cas9-edited plants were evaluated for on and off-target editing outcomes using 55x and 60x coverage whole genome shotgun sequencing data, respectively. For both potato and camelina, a prevalence of mosaic somatic edits from constitutive Cas9 expression was discovered as well as evidence of transgenerational editing in camelina. CRISPR/Cas9 editing provided negligible off-target activity compared to background variation in both species. The results from this study guide deployment and risk assessment of genome editing in commercially relevant traits in food crops.

## 1 Introduction

CRISPR/Cas9 is the most popular method for genome editing due to its versatility and simple design requirements. Originally discovered as RNA-guided endonuclease involved in an adaptive immune response in bacteria and archaea, it has now been re-engineered as a tool for sequence specific alterations in an organism’s genome (Jiang and Doudna 2017). This flexibility is particularly useful to directly improve traits or investigate gene function in crops that have lengthy breeding cycles and complex inheritance patterns. The most common CRISPR system used for gene editing in plants is derived from the CRISPR/Cas9 system in *Streptococcus pyogenes* (SpCas9) (Jinek et al., 2012). For gene editing, the SpCas9 endonuclease is targeted to a sequence using a single guide RNA (sgRNA). The sgRNA contains a user-designed RNA of ca. 20 nt that is complementary to a target region in the genome which is adjacent to a protospacer adjacent motif (PAM) of “NGG”, where N can be any nucleotide (Doudna and Charpentier, 2014). Once the targeted sequence is recognized by SpCas9, the endonuclease activity is initiated which results in a blunt ended double stranded break (DSB).

Breaks in DNA are mended through endogenous repair mechanisms which can be prone to errors. The outcomes of error prone repair mechanisms can range from single base transitions to insertions or deletions to larger structural variants like translocations (Schubert et al., 2004). It is also possible for a combination of repair pathways to act on both ends of the DSB introducing combinations of these outcomes (Vu et al., 2017). The most common repair mechanism in somatic eukaryotic cells is non-homologous end joining (NHEJ) which is divided into classical NHEJ (cNHEJ) or alternative NHEJ (aNHEJ) pathways. In cNHEJ, broken DNA ends are directly ligated back together that can result in the introduction of small insertion/deletion (InDel) often 1-3 bases long (Lieber, 2010). When microhomologies are present near the breakpoint, a common aNHEJ mechanism is microhomology-mediated end joining (MMEJ) that can introduce larger deletions, translocations, or rearrangements when DSBs are resected (McVey and Lee, 2008). Synthesis dependent strand annealing (SDSA) is homologous recombination repair pathway that can also be error prone and introduce insertions at break sites through the incomplete extension of a homologous donor (Puchta, 1998).

CRISPR/Cas9 has a reputation for being a precise way of altering genetic elements especially in plants (Peterson et al., 2016; Bessoltane et al., 2022); however, rare non-specific mutations have been documented (Zhang et al., 2014; Tang et al., 2018; Li et al., 2019; Wang et al., 2021). The frequency of off-target mutations can vary depending on delivery methods, gene editing reagent, or species (Modrzejewski et al., 2020). Careful *in silico* guided sgRNA design helps to mitigate unintended mutations by targeting highly specific sequences, but this relies on the availability of a genome sequence. Unaccounted genetic variation between the reference and transformed genotypes can result in unintended target sites, therefore a genome sequence for the transformant is preferred (Li et al., 2019; Manghwar et al., 2020). For many crops, contiguous and complete genomes are not available. Many plants have highly redundant genomes with large multicopy gene families and copy number variations, further confounded by polyploidy, which makes generating complete genome assemblies challenging. This inherent sequence similarity also increases the likelihood of genome editing dependent off-target sites that are challenging to account for without prior knowledge of the genome sequence.

*Camelina sativa* (camelina) is a sexually propagated, diploid (2n=40) with three similar sub-genomes that arose from the hybridization of an auto-allotetraploid *C. neglecta*-like species (n=13) and diploid *C. hispidia* (n=7) progenitors (Mandáková et al., 2019). Camelina is predominantly grown as an oilseed crop (Kagale et al., 2014) with a high polyunsaturated fatty acid oil composition that is prone to rancidity (Fröhlich and Rice, 2005). A well characterized target for preventing rancidity is by increasing monounsaturated fatty acid composition through the targeted removal of *fatty acid desaturase 2* (*FAD2*) which is directly involved in the desaturation of oleic acid (18:1) (Hutcheon et al., 2010). Knockout of *FAD2* in camelina using CRISPR/Cas9 has been achieved in previous studies using floral dip genetic transformation to stably integrate gene editing reagents (Jiang et al., 2017; Morineau et al., 2017).

Cultivated potato is an asexually propagated autotetraploid (2n=4x=48). Potatoes are a globally consumed food crop, and the fifth largest crop commodity produced in the world (Devaux et al., 2021; FAOSTAT). Mechanical damage to potatoes causes tuber bruising, which is a common source of food waste. Bruising is caused by oxidative browning which is controlled by *polyphenol oxidases* (*StPPO*) which is a nine member gene family in potato (Chi et al., 2014). Several studies have validated the improvement of bruising resistance in potato through the targeted suppression of *StPPO* gene members (Chi et al., 2014; González et al., 2020), including the development of commercial varieties such as Innate ™ developed by Simplot (Simplot Plant Sciences). Since potato is clonally propagated, gene edited events are recovered clonally through tissue culture or protoplast regeneration. This process is known to induce somatic mutations which have contributed significantly to the background variation in several gene editing studies (Tang et al., 2018; Li et al., 2019; Wang et al., 2021; Bessoltane et al., 2022).

This study aims to characterize the CRISPR/Cas9 based gene editing outcomes in commercially relevant traits of two crop species, potato and camelina, that have different genomic architectures and modes of reproduction. Seven Cas9 edited events targeting *StPPO* gene family members in potato generated from this study and nine events targeting *CsFAD2* generated from a previous study in camelina (Jiang et al., 2017) were analyzed through whole genome sequencing analysis. In addition, to account for variation caused by genetic transformation and tissue culture practices wild-type and empty vector transformation controls have also been analyzed. MMEJ was the primary repair pathway employed in repairing CRISPR/Cas9 DSBs in potato while cNHEJ outcomes were predominant for camelina but also produced one occurrence of a SDSA-like mechanism. The genome-wide evaluation of CRISPR/Cas9 edited transgenic events indicated that most of the genomic variation observed was independent of CRISPR/Cas9 and was either spontaneous or tissue culture induced.

## 2 Materials and methods

### 2.1 Plant material and growth conditions

The *S. tuberosum* clone DRH195 and the *C. sativa* cultivar Suneson were used in this study. DRH195 is a diploid *S. tuberosum* Phureja F1 derived from a cross between a homozygous doubled monoploid DM 1-3 516 R44 (DM) and a heterozygous diploid RH89-039-16 (RH) (Pham et al., 2020; Zhou et al., 2020). Potato plants were propagated *in vitro* using nodal cuttings in tissue culture on Murashige and Skoog (MS) medium (MS basal salts plus vitamins, 3% sucrose, 0.7% plant agar, pH 5.8) (Murashige and Skoog, 1962) and grown in culture tubes in growth chambers at 22°C with an average light intensity of 200 μmoles m^-2^ s^-1^ under a 16h photoperiod. Nine Suneson *FAD2* Cas9 edited plants were obtained from a previous study (Jiang et al., 2017); seven empty-vector control camelina lines were generated in this study (**Supplementary Table S1**). *Camelina sativa* cv. Suneson was grown in a growth chamber from seed at temperatures of 22/18°C (day/night), 40% relative humidity with a light intensity of 300 µmol m^-2^ s^-1^ under a 16h photoperiod.

### 2.2 DRH195 synthetic genome assembly

Genome assemblies and annotations for DM 1-3 516 R44 (DM) and RH89-039-16 (RHv3) were retrieved from Spud DB (Pham et al., 2020; Zhou et al., 2020; http://spuddb.uga.edu/). Whole genome sequencing data for DRH195 was retrieved from the Sequence Read Archive (SRA) of the National Center for Biotechnology Information (NCBI) under SRR4018191.

MUMmer v4.0.0’s nucmer function was used for global nucleotide alignments with the following configuration: -c 100 (Marçais et al., 2018). Global alignments were filtered using MUMmer’s delta filter to remove alignments less than 20,000 bases. SNPs between alignments were collected using MUMmer’s show-snps filtering to remove SNPs from ambiguous alignments with -C. SNPs were filtered further in R version 4.3.0 using dplyr version 1.1.2 (Wickham et al., 2023) to remove missing values and identical variants between RH haplotypes producing the final set of RH haplotype specific variants.

Whole genome shotgun reads were cleaned using Cutadapt v2.1 (Martin, 2011) to trim low-quality regions using a minimum base quality of 20 and a minimum read length of 100 bp. Picard v2.18.27 was used to convert cleaned fastq reads into an unmapped BAM using FastqtoSam and adapter sequences were marked using Mark Illumina Adapter and SamToFastq, with CLIPPING_ATTRIBUTE=XT and CLIPPING_ACTION=2 (https://github.com/broadinstitute/picard). Genomic reads were mapped to the DM reference assembly in paired-end mode, flagging secondary hits (-M), using BWA-MEM v0.7.17 (Li, 2013). Alignments were filtered to only retain properly paired reads and alignments to chromosomes 1-12 using SAMtools’ v1.7 view command (Li et al., 2009). Picard’s MergeBamAlignment was used to set metadata as well as allow for any number of insertion or deletion mutations by setting MAX_INSERTIONS_OR_DELETIONS = -1. Duplicate reads were marked using Picard’s MARKDuplicates. Reads surrounding insertion/deletions were identified and realigned using GATK’s v3.8.1 RealignerTargetCreator and IndelRealigner, respectively (McKenna et al., 2010). GATK’s Haplotypecaller v4.1.4.1 was used to call variants using default configuration. Variants were flagged using GATK v4.1.4.1 Variant Filtration using with the following expression: QD < 2.00 & MQ < 50.00 and flagged variants were removed using SelectVariants -exclude. Variants were filtered further in R version 4.3.0 using dplyr version 1.1.2 to retain variants overlapping the RH haplotype specific variant set and removing variants that did not match either of the two RH haplotype variants. A sliding window of 20 variants with 80% congruence was used to assign RH haplotype bins.

A custom script using Biopython version 1.79 in Python v3.10.4 was used to construct the DRH195 assembly (Cock et al., 2009). A gene annotation set was created for DRH195 using LiftOff version 1.6.3 (Shumate and Salzberg, 2021). Ideograms were created in R version 4.2.0 using the package chromPlot (Oróstica and Verdugo, 2016).

### 2.3 Polyphenol oxidase classification

Members of the polyphenol oxidase gene were identified by aligning previously annotated StPPO1-9 protein sequences in DMv3.4 from Chi et al. (2014) using BLASTP version 2.10.0+ with at least 90% sequence homology (The Potato Genome Sequencing Consortium, 2011; Altschul et al., 1997). Gene family members were assigned using phylogenetic inference with the Maximum-Likelihood method in MEGA X (Tamura et al., 2021).

### 2.4 Vector construction and validation

For potato, a double sgRNA construct was assembled into the pTRANS_220d binary vector using modular assembly as described by Čermák et al. (2017). We designed two sgRNAs in the ORF in conserved regions of the *StPPO* gene family in potato. Based on the potato expression data, four *StPPO*s are expressed in tubers including *StPPO*1-4, of which, *StPPO2* had the highest expression. Two single guide RNAs sgRNA1: CGCTTTGCCATATTGGAATTGGG and sgRNA2: AACACTAATGTACCGTCAAATGG were designed to target *StPPO1, StPPO2-1* and *StPPO3* using the CRISPR RGEN tools (Park et al., 2015). The two sgRNAs were cloned into pTRANS_220d using modular assembly (Čermák et al., 2017). A protoplast transient assay (Nadakuduti et al., 2019) was used to test the *in vivo* sgRNA editing activity which indicated that only sgRNA2 was active. For camelina, pTRANS_220d was modified to include DsRed2 which was used as the empty vector control. Vectors used for modular assembly and empty vector controls (https://www.addgene.org/browse/article/28189956/) were gifts from Dr. Daniel Voytas (University of Minnesota).

### 2.5 *Agrobacterium*-mediated transformation

For potato, binary vectors were electroporated into *Agrobacterium tumefaciens* strain GV3101 pMP90 (Koncz et al., 1994). Agrobacterium-mediated transformation was performed using leaf and internode explants from four-week-old tissue culture plants as described previously (Jayakody et al., 2023). Transformation events (T0 lines) were selected and transferred to MS medium supplemented with 250 mg/l cefotaxime, 300 mg/l timentin and 50 mg/l kanamycin for rooting and selection. For camelina, floral dip transformation using vacuum infiltration of floral buds was performed according to Lu and Kang (2008).

### 2.6 Transformation and event screening

For potato and camelina, DNA from transformation events was isolated from young leaves using the DNeasy Plant Mini Kit (Qiagen, Hilden, Germany). PCR for screening T-DNA insertion was carried out using the GoTaq DNA polymerase (Promega, Fitchburg, WI, United States) using primers designed to amplify an 853 bp region of Cas9 (**Supplementary Table S2**) with the following thermocycler conditions: one cycle of initial denaturation for 3 min at 95°C, followed by 34 cycles for 30 s at 95°C, 45 s at 60°C and 1 min 30 sec at 72°C and a final extension of 5 min at 72°C.

PCR amplification of *StPPO1*, *StPPO2-1* and *StPPO3* for sequencing was carried out using the NEB Q5 DNA polymerase (New England Biolabs, Ipswich, MA, United States) using primers described in **Supplementary Table S2**. PCR products were purified using QIAquick PCR Purification kit (Qiagen, Hilden, Germany) and sequenced at the Michigan State University Genomics Core. Chromatograms were analyzed for presence of indels near the target site using Synthego’s ICE CRISPR Analysis tool (Synthego Performance Analysis, 2019).

Deep sequencing of PCR amplicons was conducted on an Illumina MiSeq v2 Nano flow cell in a 2x250 nt paired-end format using amplicon sequencing primers described in **Supplementary Table S2**. Paired end reads were trimmed using Cutadapt v2.1 to remove adapters and bases with a quality score less than 20. Paired reads were joined using BBMaps’s BBMerge program (Bushnell et al., 2017). Joined reads were aligned to both haplotypes of chromosome 8 from DRH195 using BWA-MEM, marking secondary alignments. Alignments were filtered to retain only primary alignments to the amplicon’s respective target using SAMtools v1.7. Retrieved reads were analyzed using CasAnalyzer (Park et al., 2017) with a comparison range of 100, minimum frequency of 25 and a 10 base WT marker. Sanger and deep sequencing were conducted by the Michigan State University Genomics Core. Multiple sequence alignments were visualized in MEGAX using the CLUSTAL algorithm for alignment (Tamura et al., 2021).

Camelina *FAD2* CRISPR/Cas9 knockout events are described previously (Jiang et al., 2017). Briefly, F1 (sgRNA: GTCCAGTTTGTCCTCGGGTGG), R1 (sgRNA: CCACCGCAGTGTTTCAAACGCTC) and R2 (sgRNA: CCTCCCTCAGCCTCTCTCTTAC) events are either T5 or T6 generation events derived from T0 plants that had been independently transformed via agrobacterium-mediated floral dip transformation (**Supplementary Table S1**). In each lineage, a different site homologous to all *CsFAD2* homeologs was targeted by CRISPR/Cas9. The edits in the T5 and T6 events were confirmed by amplifying the F1, R1 and R2 target sites, respectively using NEB Phusion polymerase with primers listed in **Supplementary Table S2** and then digested with AvaI, BtsI and BbvCI restriction enzymes, respectively, in addition to Sanger sequencing for some lines.

### 2.7 Whole genome sequencing and library preparation

For potato, tissue was collected from leaves at 12-14 weeks from T0 events. Genomic DNA from potato and camelina events (**Supplementary Table S3**) was isolated using the DNeasy Plant Mini Kit (Qiagen, Hilden, Germany). Whole genome shotgun sequencing (WGS) libraries were prepared and multiplexed using PerkinElmer NEXTFLEX Rapid XP DNA-Seq kit, then sequenced on an Illumina NovaSeq 6000 in paired-end mode by the Texas A&M AgriLife Research Genomics and Bioinformatics Service generating 150 nt reads (**Supplementary Table S3**).

### 2.8 Variant and off-target analysis

Whole genome sequencing reads were processed and aligned as described previously. GATK Haplotypecaller v4.1.4.1 was used to call variants using default parameters. Variants with the following characteristics were removed using GATK v4.1.4.1 Variant Filtration and SelectVariants: QD < 2.00, MQ < 50.00, DP<4, DP>50, AD <4. Variants overlapping controls were removed using BEDTools v2.3.0 subtract (Quinlan and Hall, 2010). Upset plots were created in R version 4.2.0 using the package UpSetR (Conway et al., 2017). Off-target sites were identified using Cas-offinder v2.4.0 allowing up to five mismatches in the spacer sequence for canonical and non-canonical PAM sites (Bae et al., 2014). SPAdes v3.15.5 was used for *de novo* assembly (Prjibelski et al., 2020).

## 3 Results

### 3.1 DRH195 synthetic genome construction

To facilitate WGS analysis for detecting off-target gene editing in potato, a synthetic genome assembly was constructed for DRH195. First, a set of variants that could discern the two haplotypes in the heterozygous RH clone were identified by aligning chromosomes from both haplotypes independently to the respective chromosome in DM. SNPs between alignments were identified and filtered to retain unambiguous variants at shared locations that were unique to each RH haplotype. Then, to assign the RH haplotype inherited in DRH195, alternate alleles called from DRH195 WGS aligned to DM were compared to the RH haplotype specific variant set. Haplotype bins were assigned using a sliding window of 20 variants with 80% congruency.

**Figure 1:**
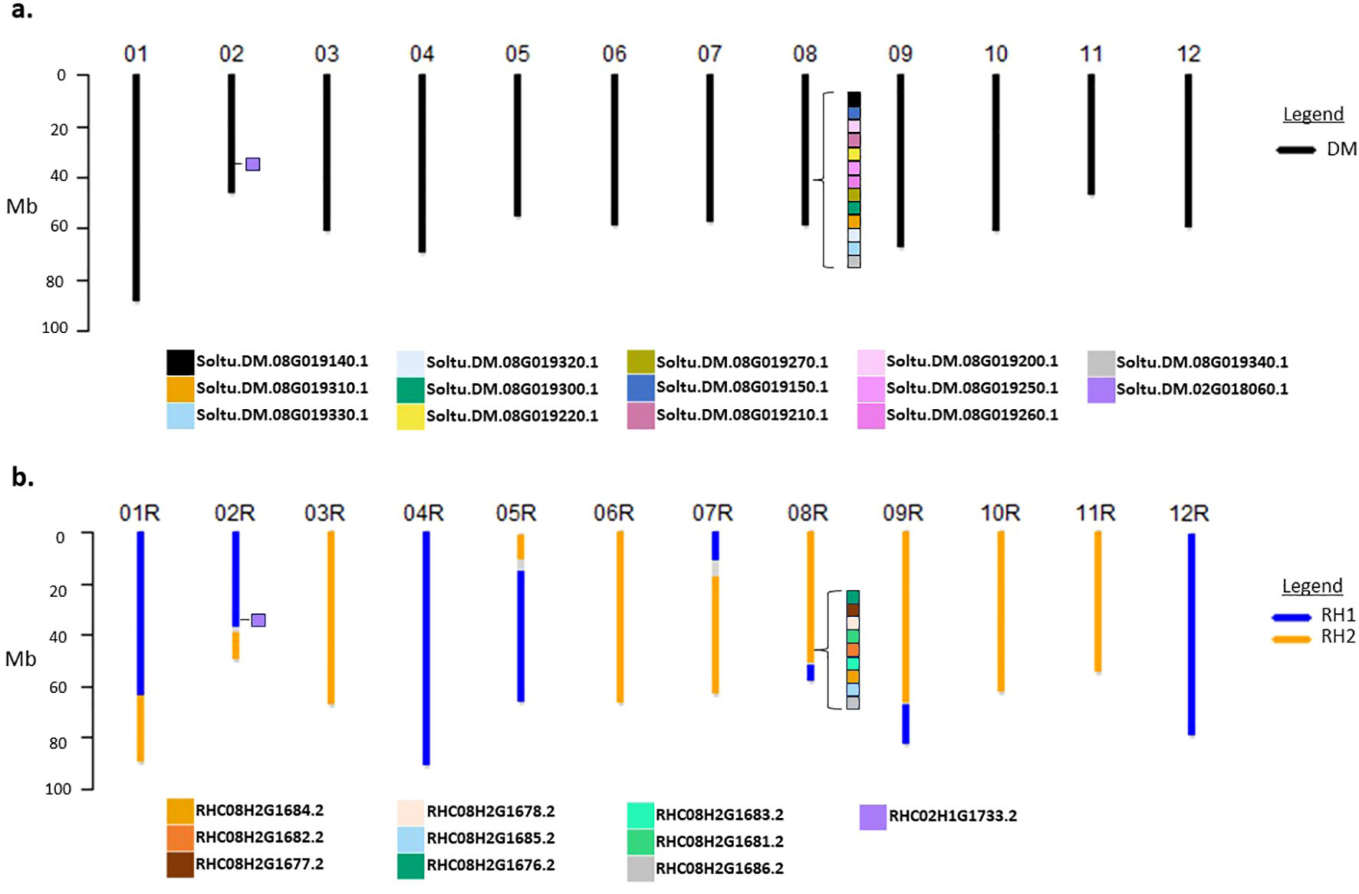
Ideogram representation of DRH195 haplotype assignment of **(A)** DM and **(B)** RH haplotypes in synthetic genome assembly with position of *StPPO*s marked by colored squares on chromosomes 2 and 8 indicated by protein IDs from **Supplementary Table S4**. Gaps in DRH195 assembly are indicated on the ideogram in gray. *StPPOs, Polyphenol oxidases* in *S. tuberosum*.

A synthetic chromosome scale assembly for DRH195 was then constructed by assuming all chromosomes from the homozygous DM were inherited. For the RH haplotype, the assigned haplotype sequences were extracted from the RH genome assembly. Phasing of the haplotype inherited from RH uncovered recombination events on chromosomes 1,2,5,7,8 and 9, while the remaining chromosomes retained the entirety of one of either RH haplotypes (**Figure 1**).

### 3.2 Classification of *Polyphenol oxidases* in potato DRH195

A previous study annotated all members of the *polyphenol oxidase* (*PPO*) gene family present in potato through a genome wide survey using the DMv3.4 reference assembly (Chi et al., 2014). The protein models designating StPPO1-9 from that study were used for phylogenetic inference to assign the homologous sequences in DMv6.1 and RHv3 (**Supplementary Table S4**). This uncovered an additional *PPO*-like sequence in DMv3.4 that had not previously been described that was most like *StPPO5* in sequence (*StPPO5-2*). The more contiguous DMv6.1 assembly revealed one additional *StPPO3* and three additional *StPPO7* copies that were not present in the DMv3.4 reference genome.

A single copy of *StPPO1-9* was identified for each gene family member in haplotype 1 of the RHv3 assembly. The second haplotype of RHv3 contains two full length copies of *StPPO2* like sequence in addition to two truncated *StPPO2* like sequences, however, *StPPO1*, *StPPO5, StPPO6* or *StPPO7* like sequences were absent (**Supplementary Table S4**; **Figure 1**). The region on chromosome 8 where the *StPPO1-8* are present on RH haplotype 2 was inherited in DRH195 (**Figure 1**). Although only one *StPPO1* like sequence was identified in DMv6.1 and none in the RHv3 haplotype 2, chromatograms from sequencing *StPPO1* in WT DRH195 indicated the presence of a second allele (**Supplementary Figure S1**). To recreate the entire open reading frame of the second *StPPO1* like sequence, a consensus sequence was created using the alternate alleles called from WGS of WT DRH195 aligned to *StPPO1* in the DM assembly. This sequence retains the identical sgRNA2 target site and PAM sequence.

### 3.3 CRISPR-Cas9 based targeted mutagenesis of *StPPO* and screening of gene-edited events in potato

DRH195 was genetically transformed using CRISPR constructs with sgRNAs targeting *StPPOs* and the empty vector control. Kanamycin resistance was used for selecting transgenic events and PCR amplification of Cas9 was used to confirm T-DNA integration. Only events with clear kanamycin resistance and PCR confirmation were selected for further analysis. Five T0 empty vector control and 27 T0 events from CRISPR construct were generated for potato in this study. However, only 7 of the 27 T0 events had confirmed insertion/deletion mutations in at least one *StPPO* target site (**Supplementary Table S5**).

**Figure 2:**
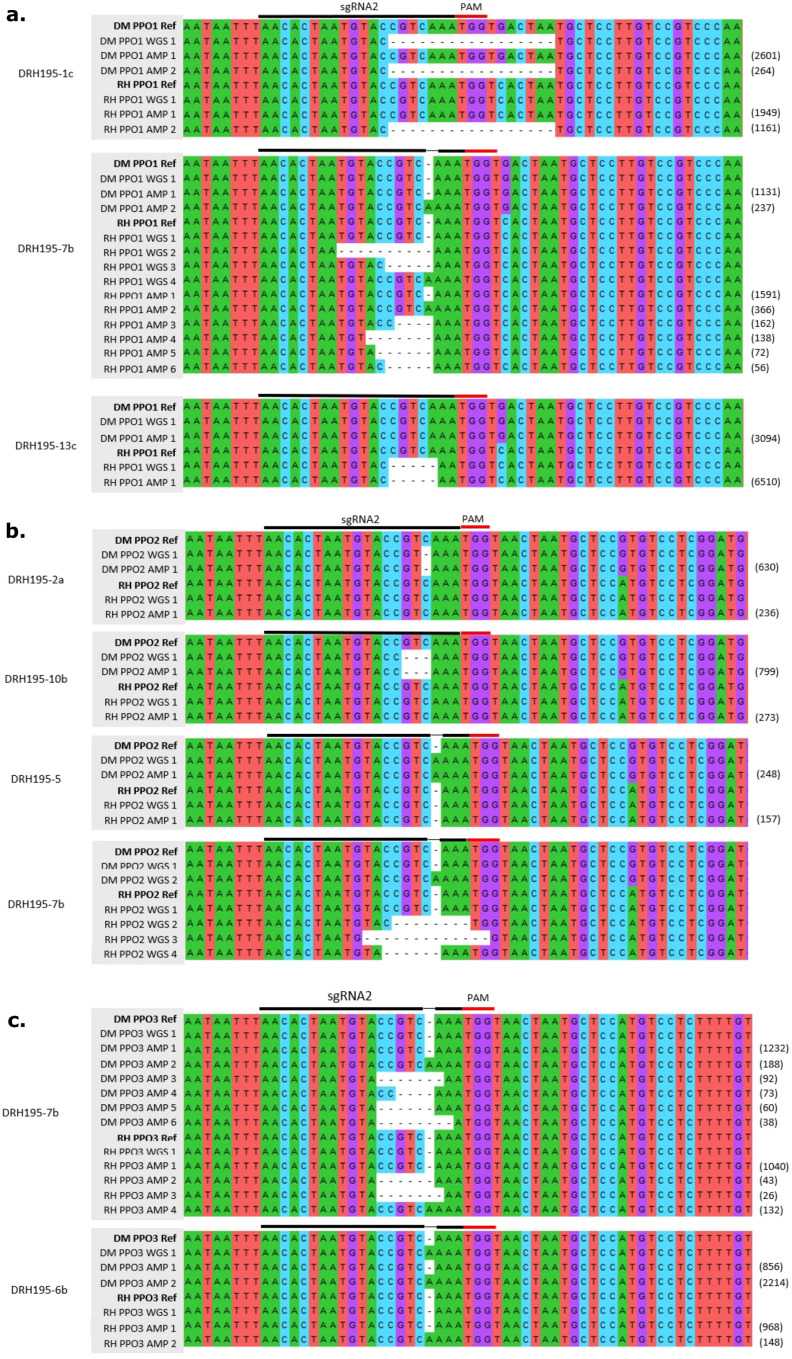
Multiple sequence alignment of variants detected at on-target sites for sgRNA2 from whole genome sequencing (WGS) and deep sequencing (AMP) at **(A)** *StPPO1*, **(B)** *StPPO2* and **(C)** *StPPO3* sgRNA2 site in DRH195 Cas9 events. Values in parentheses are the number of reads supporting a deep sequenced amplicon.

Both alleles of *StPPO1*, *StPPO2-1*, and *StPPO3* are targeted by sgRNA2 in DRH195 and were screened for mutations in T0 plants (**Figures 2A–C**). Given this abundance and the sequence homology between *StPPO* gene family members in potato, a semi-nested PCR was used to differentiate *StPPO* paralogs and alleles from each other. The first round of PCR used primers specific to both alleles of *StPPO1*, *StPPO2-1* or *StPPO3* in DRH195 (**Supplementary Table S2**). To facilitate Sanger sequencing of amplicons, the second round of PCR tagged the M13 forward sequence to the 5’ end of the forward primer which was located ca. 300 bp upstream from the predicted sgRNA2 edit site. Indels were identified at target sites using Synthego’s ICE algorithm which identifies the positions where Sanger traces are discordant between wildtype and mutated amplicons (Conant et al., 2022; Synthego Performance Analysis, 2019).

To verify allelic representation, the same purified PCR products for Sanger sequencing were deep sequenced using Illumina sequencing. Chimeric PCR products indicative of mixed template amplifications was observed in events DRH195_1c *StPPO1* and DRH195_6b *StPPO3* from amplicon sequencing (**Figures 2A, C**). Chimeric PCR products were also seen in wild-type amplicon controls supporting that these are PCR artifacts and not recombination events. No T0 event had an edit in all alleles of the targeted *StPPOs*. In all cases except for event DRH195_7b, only one allele of one paralog was edited. Mosaic edits were observed in at least one allele of *StPPO1* and *StPPO3* for DRH195_7b (**Table 1**). Although ICE could not differentiate between alleles, the predicted indel sizes were congruent with the results from deep sequencing (**Table 1**).

**Table 1:** Comparison of variant detection methods for identifying CRISPR/Cas9 gene-editing outcomes at sgRNA2 on-target sites in DRH195 events.

	ICE^a^		
Event	PPO1	PPO2-1	PPO3
DRH195_1c	1(-17)/2	0/2	0/2
DRH195_6b	0/2	0/2	1(+1)/2
DRH195_5	0/2	1(+1)/2	0/2
DRH195_7b	1(+1,-3,-6)*/2	0/2	1(0, + 2,+11,-19,-23,-29,-22,-12,+1,-27,-10,-18,-19)*/2
DRH195_10b	0/2	1(-3)/2	0/2
DRH195_13c	1(-5)/2	0/2	0/2
DRH195_2a	0/2	1(-1)/2	0/2
	Deep-Seq		
Event	PPO1	PPO2-1	PPO3
DRH195_1c	DM(0,-17)**/RH(0,-17)**	NA	NA
DRH195_6b	NA	NA	DM(0, + 1)**/RH(0, + 1)**
DRH195_5	NA	DM(+1)/RH(0)	NA
DRH195_7b	DM(0, + 1)*/RH(0, + 1,-6,-5,-4,-3)*	NA	DM(0, + 1,-3,-5,-6,-7)*/RH(0, + 1,-5,-6)*
DRH195_10b	NA	DM(-3)/RH(0)	NA
DRH195_13c	DM(0)/RH(-5)	NA	NA
DRH195_2a	NA	DM(-1)/RH(0)	NA
	WGS		
Event	PPO1	PPO2-1	PPO3
DRH195_1c	DM(-17)/RH(0)	DM(0)/RH(0)	DM(0)/RH(0)
DRH195_6b	DM(0)/RH(0)	DM(0)/RH(0)	DM(+1)/RH(0)
DRH195_5	DM(0)/RH(0)	DM(+1)/RH(0)	DM(0)/RH(0)
DRH195_7b	DM(0)/RH(0)	DM(0, + 1)*/RH(0,-5,-7,-12)*	DM(0)/RH(0)
DRH195_10b	DM(0)/RH(0)	DM(-3)/RH(0)	DM(0)/RH(0)
DRH195_13c	DM(0)/RH(-5)	DM(0)/RH(0)	DM(0)/RH(0)
DRH195_2a	DM(0)/RH(0)	DM(-1)/RH(0)	DM(0)/RH(0)

a 0/2 = two WT sequences; 1/2= one edited and one WT sequence; 2/2= two edited sequences.

DM=DM allele; RH=RH allele.

*mosaic edits.

**chimeric PCR artefact.

Value in parentheses represents size of insertion (+) or deletion (-).

### 3.4 Targeted mutagenesis of *Fatty acid desaturase* using CRISPR-Cas9 in *Camelina sativa* and screening of edited events

A single copy of *CsFAD2* is present on each of the homoeologous chromosomes 1, 15 and 19 designated by the following Suneson gene model IDs Camsa.SUN.01G012720.1, Camsa.SUN.15G013420.1 and Camsa.SUN.19G013580.1, respectively. Jiang et al. (2017) designed three independent sgRNAs for F1, R1 or R2 events, each of which targeted the three homeologs of FAD2. Suneson was transformed with the same empty vector control construct as potato but modified to include *DsRed2* marker for seed selection (**Supplementary Table S1**). PCR amplification of *DsRed2* was used to confirm T-DNA integration and PCR positive events were phenotyped for DsRed-positive seed and selected for further analyses. Seven empty vector controls for camelina were generated in this study (**Supplementary Table S1**). On-target editing was confirmed for Suneson *CsFAD2* KO events Jiang et al. (2017) using the following restriction enzyme digestions: *Ava*I for F1 sites, *Bts*I for R1 sites and *Bbv*CI for R2 sites. Events with resistant bands indicated disrupted restriction sites due to Cas9-editing and were selected for WGS analysis (**Supplementary Table S1**).

### 3.5 Whole genome sequencing of gene-edited events and analysis of editing outcomes

For potato, the DRH195 synthetic genome assembly was used as the reference for WGS analysis. Across the samples, an average of 55x coverage for the haploid genome was obtained, except for event DRH195_2a which had 91x coverage (**Supplementary Table S3**). Coverage was normalized for event DRH195_2a by taking a random subsample of reads using the median coverage from potato events, 58x. An average of 99.58% of reads mapped to the reference genome sequence for all samples. WGS analysis supported the assignment of variants identified through deep sequencing at the target site for the T0 events DRH195_13c, DRH195_2a, DRH195_10b and DRH195_5, with no additional variants at other on-target sites (**Table 1**, **Figure 2**). Deep sequencing of Cas9 event DRH195_7b indicated a mosaic edit in the RH and DM alleles of *StPPO1* and *StPPO3*, but no variants were called at these locations from WGS (**Table 1**). Although the expected *StPPO1* and *StPPO3* variants were not called, there were multiallelic variants, indicative of mosaic edits in both alleles of *StPPO2-1* from WGS (**Figure 2B**). These results are consistent with T0 regenerated plants of the vegetatively propagated potato being a chimera of edited and wild-type alleles. On-target variations in potato ranged from a 17-base deletion to 1 base insertion, all within the seed sequence (**Table 1**, **Figure 2**). Larger deletions and more variable in sizes were more commonly detected in potato, the largest being a 17bp deletion (**Table 1**, **Figure 2A**). This characteristic combined with the presence of microhomology at target sites indicates that MMEJ was the repair mechanism employed.

For camelina, the Suneson genome assembly was used as the reference (Fang et al., 2023) and~60x coverage was obtained with an average of 99.76% of reads mapping to the reference. Only variants that were unique to each CRISPR/Cas9 edited event from the respective wild-type and empty vector controls were considered for NHEJ outcomes.

**Figure 3:**
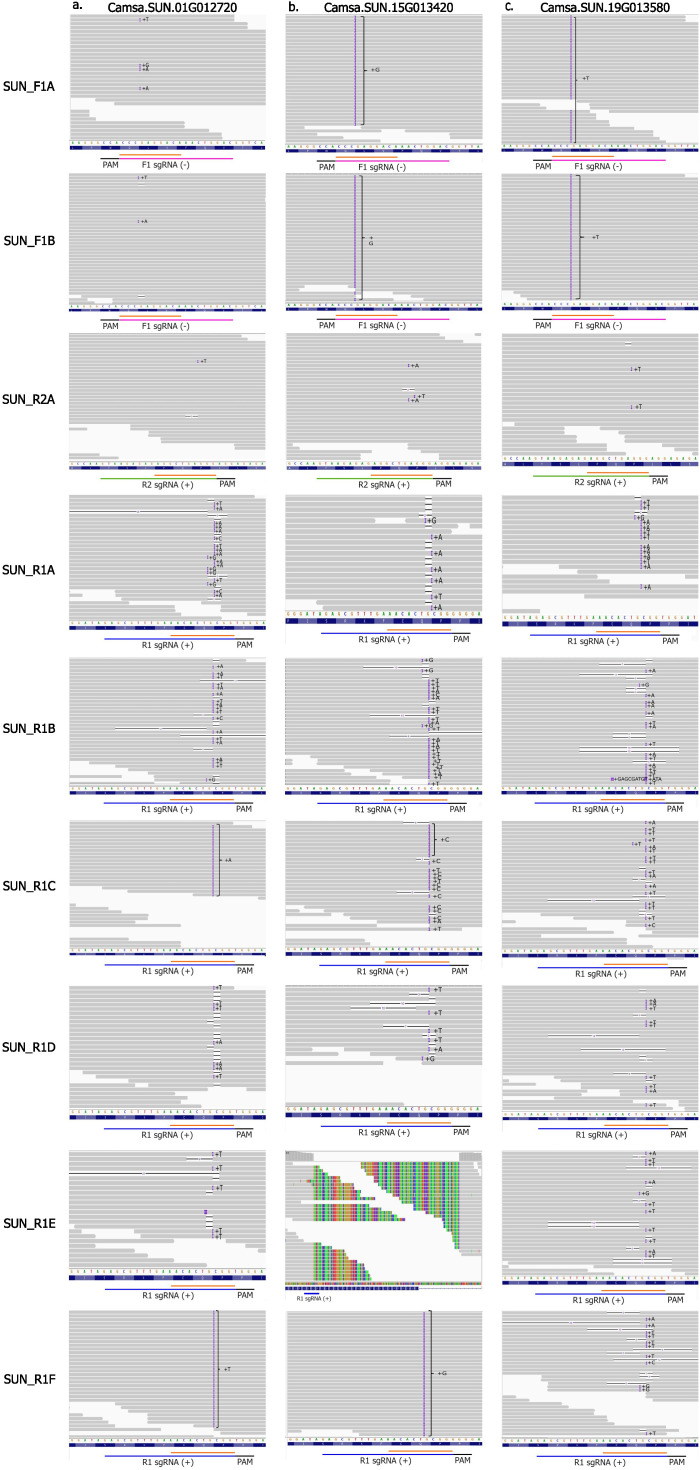
Whole genome sequencing read alignment visualized in the Integrative Genome Viewer (IGV) for camelina Cas9 events at their respective target sites in the three FAD2 homeologs **(A)** Camsa.SUN.01G012720, **(B)** Camsa.SUN.15G013420 and **(C)** Camsa.SUN.19G013580.Deletions are represented by black lines and insertions by the purple boxes. Sequences for F1, R1 and R2 spacers are indicated by a pink, blue and green line, respectively. Seed sequences of the spacers are indicated by an orange line. Values in parentheses indicate strand.

No T5 or T6 camelina events had homozygous edits in all *CsFAD2* homeologs suggesting that a complete loss of function of this trait may be lethal. All R1 events at their target sites had evidence of either fixed or a mosaic editing at all sites (**Figure 3**). Transgenerational editing (TGE), which is continued editing throughout multiple generations, was observed in camelina events due to T-DNA integration and constitutive expression of CRISPR/Cas9 reagents as reported earlier (Impens et al., 2022; Jiang et al., 2017). In R1 events, a mosaic of wild-type and mutant reads were observed at most target sites. Jiang et al. (2017) also detected TGE at target sites into the T3 generation for R1 events. In this study, we observed that TGE continued into the T5 and even T6 generations in R1 events. Several homozygous sites had 1bp on-target insertions within the seed sequence, first 10 nts upstream of the 3’ end of the sgRNA (**Figure 3**). These sites remained unaltered by additional TGE, supporting previous reports of CRISPR/Cas9 specificity and preference toward editing sites without variation in seed sequences (Liu et al., 2016). The rate of TGE varied between the F1, R1 and R2 events, with R1 events having the highest mutation rate. This is consistent with results reported by Jiang et al. (2017). Only a few reads suggest TGE in events at the F1 and R2 sites although a higher read depth would be needed to distinguish from sequencing error (**Figure 3**).

Evidence of a complex variant was observed at the target site in Camsa.SUN.15G013420 in the T5 event SUN_R1E, as seen by clipped reads with partial homology to the reference (**Figure 3B**). To identify the full sequence composition of this variant, the WGS reads were used to create a *de novo* contig assembly that indicates 195 bases were replaced by a 166 bp insertion. This variant was then confirmed through cloning and sequencing. This insertion has no significant homology to any other region in the genome or to the vector. This variant is suggestive of a SDSA like repair mechanism in combination with cNHEJ.

Most on-target variants detected in camelina were 1bp insertions 3 bases upstream of PAM site at the predicted cut site for Cas9. Furthermore, no insertions larger than 1bp were detected in either species in this study. The preference toward one base pair insertion in camelina was also described in Jiang et al. (2017) where there was a noticeable enrichment in insertions over deletions at target sites with 99% of insertions being single nucleotide. Insertions of 1-3 bp are characteristic of cNHEJ mediated repair indicating a preference toward cNHEJ in camelina at all three target sites.

### 3.6 Off target effects of gene-editing by CRISPR-Cas9 was compared to Cas9-independent transgenic events in potato and camelina by whole genome sequencing analyses

Sequence variation was observed between wild-type controls and the DRH195 and Suneson reference assemblies (**Table 2**). For potato, there was a larger proportion of SNP variants compared to indels, with an average of 57,344 SNPs to 18,109 indels per transgenic event. This contrasts variation seen in camelina which had a larger proportion of indel variants to SNPs with an average of 4,041 SNPs to 36,379 indels per transgenic event. This trend is also seen in wild-type controls compared to the reference, with 5x more SNP to indel variants in potato versus 5x more indel to SNP variants in camelina. Most indel variants in camelina events are single nucleotide insertions or deletions (**Supplementary Figure S2**). The consistency of indel mutations across all events and controls indicates that this variation is more likely related to common sequencing errors observed in Oxford nanopore derived genome assemblies (Oxford Nanopore Technologies, 2024) relative to the PacBio-derived RH assembly which is 50% of the overall DRH195 genome assembly.

**Table 2:** Summary of SNP and indel variants called from gene-edited potato and camelina events using whole genome sequencing.

		Lines vs Ref		Lines vs Ref+WT		Lines vs Ref+WT+EV	
Line name	Species	snp	indel	snp	indel	snp	indel
DRH195 WT-1	*Solanum tuberosum*	501909	99938	–	–	–	–
DRH195 WT-2	*Solanum tuberosum*	623932	112849	–	–	–	–
DRH195_EV_3c	*Solanum tuberosum*	628922	115792	53229	16507	–	–
DRH195_EV_7b	*Solanum tuberosum*	679651	129509	73535	23479	–	–
DRH195_EV_11a	*Solanum tuberosum*	665378	125768	66495	21392	–	–
DRH195_EV_12a	*Solanum tuberosum*	670217	126751	70317	22223	–	–
DRH195_EV_14c	*Solanum tuberosum*	663281	124842	65160	20834	–	–
DRH195_1c	*Solanum tuberosum*	593124	106633	39843	12208	9935	4537
DRH195_6b	*Solanum tuberosum*	644539	120982	55313	18575	14567	7539
DRH195_5	*Solanum tuberosum*	605266	109563	42269	12952	10091	4657
DRH195_7b	*Solanum tuberosum*	649852	122111	56783	19012	14622	7675
DRH195_10b	*Solanum tuberosum*	639305	119509	51832	17211	12859	6744
DRH195_13c	*Solanum tuberosum*	679854	129093	75171	23521	22886	10249
DRH195_2a	*Solanum tuberosum*	500678	84760	38183	9400	15259	4106
Suneson WT3-1	*Camelina sativa*	12226	60612	–	–	–	–
SUN_DsRed_1.1a.1	*Camelina sativa*	10902	60673	4449	36189	–	–
SUN_DsRed_1.1c.1	*Camelina sativa*	14749	59577	6364	35750	–	–
SUN_DsRed_3.1a.1	*Camelina sativa*	14341	58113	6201	34412	–	–
SUN_DsRed_3.1b.1	*Camelina sativa*	10888	60082	4519	35631	–	–
SUN_DsRed_5.1.1	*Camelina sativa*	12600	60115	5389	35915	–	–
SUN_DsRed_6.1a.1	*Camelina sativa*	10795	60550	4595	36039	–	–
SUN_DsRed_6.1b.1	*Camelina sativa*	13113	57772	5387	34435	–	–
SUN_F1A	*Camelina sativa*	10097	60871	3652	36942	1001	11238
SUN_F1B	*Camelina sativa*	5503	62471	2550	37676	893	10043
SUN_R2A	*Camelina sativa*	6756	60903	2655	36731	763	10184
SUN_R1A	*Camelina sativa*	8488	61192	3287	36763	971	10423
SUN_R1B	*Camelina sativa*	6579	62447	2709	37514	900	10246
SUN_R1C	*Camelina sativa*	9168	61580	3302	37049	964	10845
SUN_R1D	*Camelina sativa*	7698	62843	2875	38065	901	10839
SUN_R1E	*Camelina sativa*	11488	58609	4039	34822	901	10592
SUN_R1F	*Camelina sativa*	6904	63168	2693	38139	832	10599

Off-target sites containing up to 5 mismatches in the target sequence for canonical (NGG) and non-canonical (NGA, NAG) PAMs were identified in the DRH195 and Suneson assemblies using Cas-Offinder (Bae et al., 2014). Only variants that were unique to CRISPR/Cas9 edited events compared to the empty vector and wild type controls were considered for off-target analysis. In potato, two canonical NGG off-target sites identical to sgRNA2 were identified, both of which were on chromosome 8 of the RH haplotype. The first off-target site overlapped with the CDS of *StPPO2-2* and the second off-target site overlapped with the 5’ UTR of the following gene model RHC08H2G1680.2. RHC08H2G1680.2 shares partial sequence homology to the 3’ end of the adjacent gene model *StPPO4-3* suggesting that this may also belong to *StPPO* gene family. Analysis of variants across all 7 events indicated no edits in either of these off-target sites (**Table 3**).

**Table 3:** Summary of canonical NGG off-target sites for sequences with equal to or less than 5 mismatches as detected by Cas-Offinder for potato and camelina Cas9 events.

	Mutations/No. NGG Sites					
Line	mismatch =0	mismatch =1	mismatch =2	mismatch =3	mismatch =4	mismatch =5
DRH195_1c	0/2	NA	0/3	0/9	0/97	2/787
DRH195_6b	0/2	NA	0/3	0/9	0/97	0/787
DRH195_5	0/2	NA	0/3	0/9	0/97	0/787
DRH195_7b	0/2	NA	0/3	0/9	0/97	0/787
DRH195_10b	0/2	NA	0/3	0/9	0/97	0/787
DRH195_13c	0/2	NA	0/3	0/9	0/97	2/787
DRH195_2a	0/2	NA	0/3	0/9	0/97	0/787
SUN_F1A	NA	NA	0/1	0/19	0/273	0/1611
SUN_F1B	NA	NA	0/1	0/19	0/273	0/1611
SUN_R2A	NA	NA	NA	0/13	1/115	9/1275
SUN_R1A	NA	NA	NA	NA	0/9	0/147
SUN_R1B	NA	NA	NA	NA	0/9	0/147
SUN_R1C	NA	NA	NA	NA	0/9	0/147
SUN_R1D	NA	NA	NA	NA	0/9	0/147
SUN_R1E	NA	NA	NA	NA	0/9	0/147
SUN_R1F	NA	NA	NA	NA	0/9	0/147

NA, no off-target site detected.

In the remaining canonical and non-canonical PAM off-target sites in potato, less than 0.1% of putative off-targets in any event contained a variant (**Table 3**; **Supplementary Table S6**). Two canonical NGG off target sites with five mismatches in the target sequence contained a variant in events DRH195_1c and DRH195_13c, but manual inspection of alignments showed the same SNP variant was shared between the two transgenic events suggesting this as a tissue culture induced somatic variant. Seven non-canonical NGA sites with five mismatches in the target sequence contained a variant across all potato events, but only four of the variants were unique between samples. Manual inspection of alignments showed that all variants were SNPs which are not a common outcome of Cas9 dependent editing. Furthermore, the SNPs were supported by reads present in other samples or controls, but with an allele fraction below the threshold to be called a variant from WGS analysis. Therefore, these variants are also classified as background and not Cas9 dependent off target edits, resulting in no substantial evidence for off-target editing in potato.

In camelina, no additional canonical PAM off target sites with exact matches to the F1, R1 or R2 target sites were identified (**Table 2**; **Supplementary Table S6**). Of the remaining canonical and non-canonical PAM off-target sites with up to five mismatches, only event SUN_R2A had putative off-targets variants, with less than 0.8% of off target sites containing a variant representing less than 0.7% of the total genetic variation in this event. The majority of these off-targets were in non-canonical NGA PAM sites with five mismatches (**Supplementary Table S6**). Contrary to the results in potato, most of the off-target variants were short deletions. Out of the 77 off-target variants detected in SUN_R2A, 59 were 2bp deletions. Indels were the most common spontaneous variant type identified across all events and controls for camelina in this study (**Table 2**). A total of 11% of all indels in SUN_R2A were 2bp deletions which was the third most frequent variant type in this event (**Supplementary Figure S2**). As mentioned previously, Cas9 editing most often resulted in 1bp insertions for these camelina events (Jiang et al., 2017). Together, this suggests that the indels present at these putative off-target sites may likely be attributed to sequencing errors in the reference assembly, although further investigation into these sites is necessary to determine which variants are *bona fide* Cas9 dependent off-target mutations.

**Figure 4:**
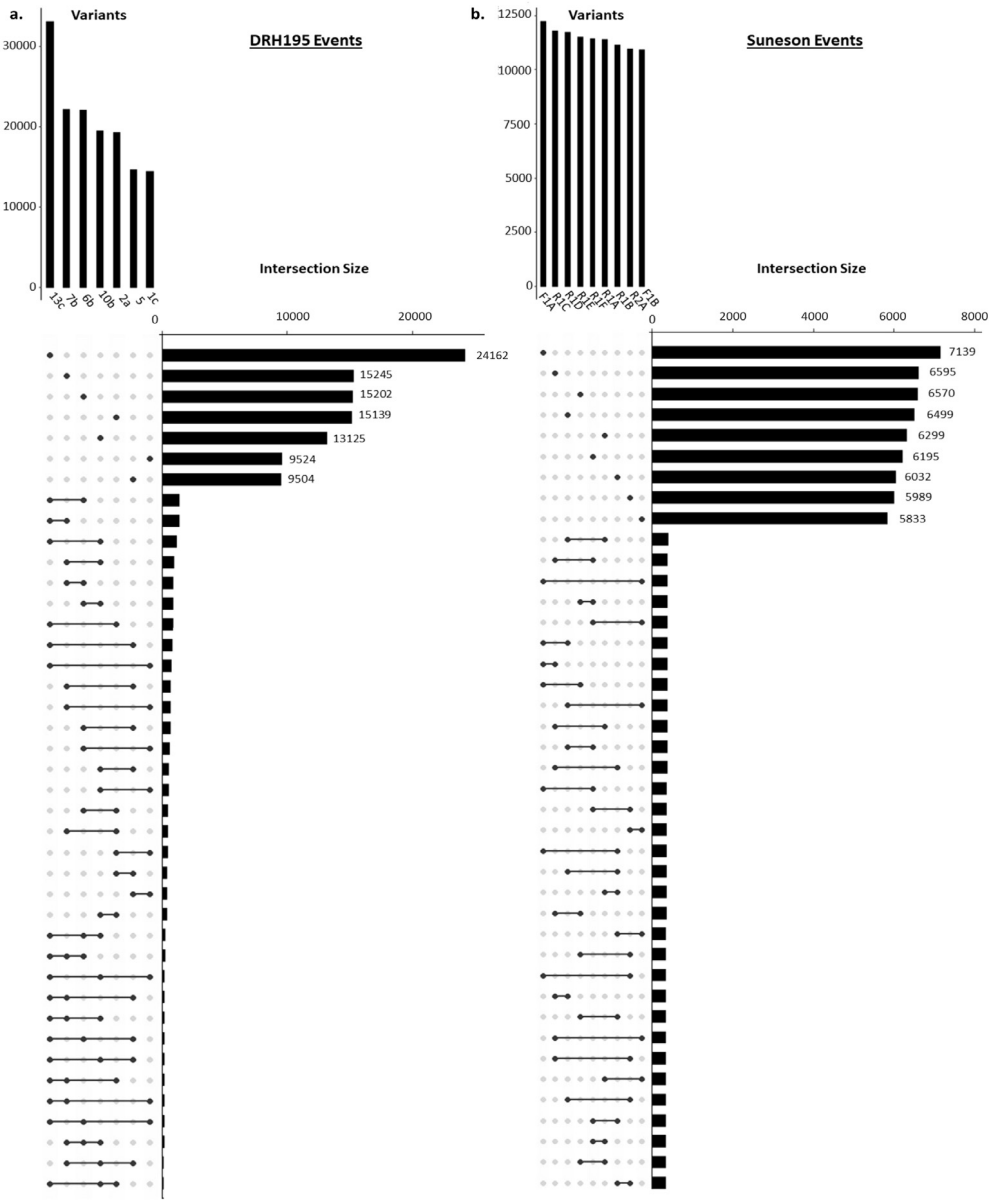
Upset plot of WGS variant intersections for **(A)** DRH195 potato *StPPO* CRISPR KOs and **(B)** Suneson camelina *CsFAD2* CRISPR KOs. Variants represented are unique to the event compared to the wildtype and empty vector controls. Upset plot was sorted from largest number of variants to smallest. The vertical bars represent total variants per event. The black dots represent the event(s) being compared to the horizontal black bars which represent the variant count for each set. Connected dots by black lines represent variants shared between events.

**Figure 5:**
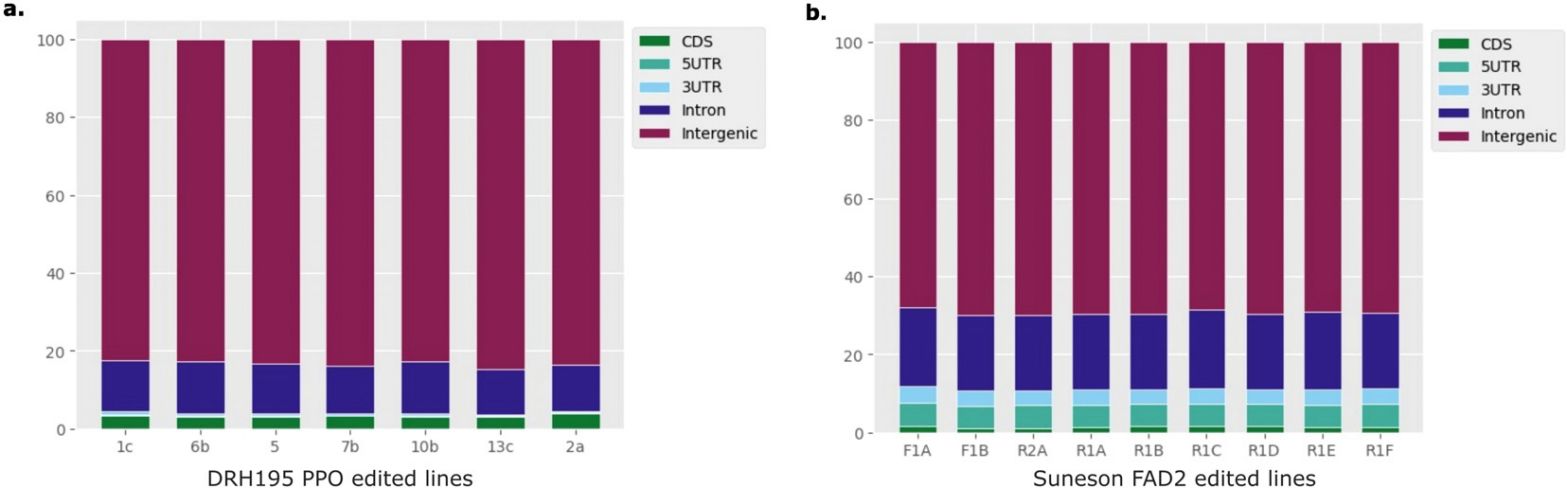
Percentage of variants overlapping 5'UTR, 3'UTR, exon, intron or intergenic regions in **(A)** DRH195 potato *StPPO* CRISPR KOs or **(B)** Suneson camelina *CsFAD2* CRISPR KOs.

**Figure 6:**
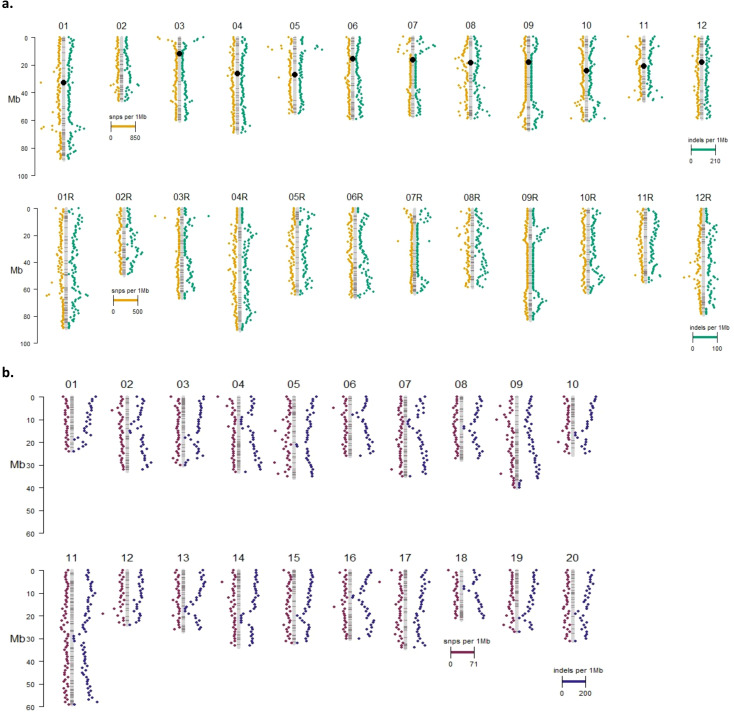
Insertion and deletion variant landscape for **(A)** DRH195 and **(B)** Suneson CRISRPR/Cas9 events. Black circles represent locations of centromeres. DRH195 chromosomes designated with 'R' represent the RH haplotype.

Most variants identified in the potato and camelina Cas9 events were outside of the putative on- or off-target sites and were unique to each event (**Figure 4**, **Table 2**). In both species most variants were intergenic (**Figure 5**). Generally, variants are called in the euchromatic chromosome arms, but on chromosome 8 in DRH195, variants were called across the entire chromosomes, including the heterochromatic region across all CRISPR/Cas9-edited events (**Figure 6A**). The positions of the centromeres are not available for Suneson, but on nearly every chromosome there is a region where the number of indel variants dips, which may be a suggestion to the position of the centromeres (**Figure 6B**). In Suneson, the SNPs were called across the entire chromosome, whereas the indel mutations are localized in the presumed euchromatic regions. Overall, potato accumulated more Cas9 independent variants mostly likely due to tissue culture induced mutations than the seed propagated camelina (**Figure 4**, **Table 2**).

## 4 Discussion

The results described here support previous reports that CRISPR/Cas9 editing contributes negligible, if any, mutational load compared to the somatic variants produced from tissue culture or spontaneous mutations from sexual propagation (Peterson et al., 2016; Tang et al., 2018; Li et al., 2019; Wang et al., 2021; Bessoltane et al., 2022). In other unbiased studies using whole genome sequencing to detect off target mutations, variants were predominantly tissue culture or spontaneous induced (Zhang et al., 2014; Tang et al., 2018; Li et al., 2019; Wang et al., 2021; Bessoltane et al., 2022). In several of these studies, *bona fide* off target editing was detected, mainly at sites that contained 1 or 2 SNPs outside of the seed sequence in the spacer (Zhang et al., 2014; Tang et al., 2018; Li et al., 2019; Wang et al., 2021). These off-target sites could be detected by off-target prediction software, reinforcing the importance of careful sgRNA design that incorporates *in silico* off-target prediction. In our study, variants in off-target sites were detected in two potato and one camelina event, but only at sites with 4-5 SNPs in the target sequence and with variants that are more like common background variants.

Cas9 dependent off-target variation continued to be negligible even in camelina events with constitutive Cas9 expression into the sixth generation with a noticeable TGE preference towards on-target sites. A notable source for unintended genome editing effects in plants is unexpected variation between the reference genome and the edited individual. Particularly when targeting gene families, it is likely to encounter unanticipated on-target sites. This was seen in potato with on-target matches present in the RH haplotype that were not identified in the DM potato reference genome. In addition, an on-target site that was not accounted for in either haplotype of the DRH195 assembly was also identified through targeted sequencing. Targeted sequencing using third generation sequencing methods can help to resolve ambiguities in genomic regions with many paralogous sequences.

We described the prevalence of mosaic editing in T0 potato as well in TGE in T5 and T6 camelina Cas9 events. Although mosaic edits are a common genome editing outcome, no WGS study evaluating Cas9 editing in plants has attempted to characterize mosaic edits. Notable challenges exist in distinguishing somatic mutations from WGS analysis, as somatic variants require high read coverage for reliable variant calling which can be prohibitively expensive for routine use. Deep sequencing of an individual target may be preferred, although this may be challenging in highly homologous sequences such as the *CsFAD2* homeologs in camelina or *StPPO* gene family in potato. Improved methods for screening rare somatic variants in plants are necessary. For a seed propagated crop like camelina, the impacts of mosaicism can be overcome through the fixation of mutated alleles in subsequent generations where Cas9 has been segregated out. However, for vegetatively propagated crops like potato where clonal identity is required this approach is not feasible. This underpins a major challenge in genome editing of vegetatively propagated crops when using traditional stable genetic engineering approaches. In practical applications, transient approaches such as direct delivery of CIRSPR/Cas9 cassettes as ribonucleoprotein may be preferred to mitigate the chance of mosaic edits.

Targeting multiple genes with one sgRNA resulted in no complete knock outs detected in T0 events in potato (this study) or camelina (Jiang et al., 2017). The difference in repair mechanisms employed at potato and camelina on-target editing sites is the outcome of gRNA design and genome structure. In the case of potato, microhomologies in the spacer sequences resulted in a bias toward MMEJ repair compared to a cNHEJ response in camelina which lacked microhomologies. Furthermore, mutation caused by SDSA was only detected in camelina suggesting the presence of a homologous repair template within camelina.

In sexually propagating individuals, transgenerational editing can be leveraged to select for events with homozygous edits in subsequent generations, but not in the case of asexually propagated species like potato. There is a bottleneck in editing efficiency when working with polyploids or multicopy gene families, which could be overcome through continued effort toward species specific optimization of vectors and transformation methods (Zhou et al., 2023).

In conclusion, CRISPR/Cas9 is specific to target sites in both camelina and potato but genotype specific whole genome sequencing and *in silico* off target detection, should be incorporated with target design to avoid unanticipated target sites and aid in the interpretation of common assembly errors.

## Data Availability

The raw genomic sequences are available in the NCBI SRA database under BioProject PRJNA1137361. The DRH195 synthetic genome assembly and annotation are available in the Dryad Digital Repository (doi: https://doi.org/10.5061/dryad.n5tb2rc4n).

## Supplementary material

Supplementary Figure 1 Chromatogram of PPO1 in wildtype DRH195. Orange arrow indicates presence of mixed template.

Supplementary Figure 2 Summary of indel lengths from -4 to 4 basepairs for all camelina events.
