# Supplementary figures and images for: Genome-wide evaluation of gene editing outcomes using CRISPR/Cas9 in seed propagated Camelina sativa and vegetatively propagated Solanum tuberosum
Source: Front Plant Sci. 2024 Nov 26;15:1496861. doi: 10.3389/fpls.2024.1496861 (PMC11628256; doi:10.3389/fpls.2024.1496861)

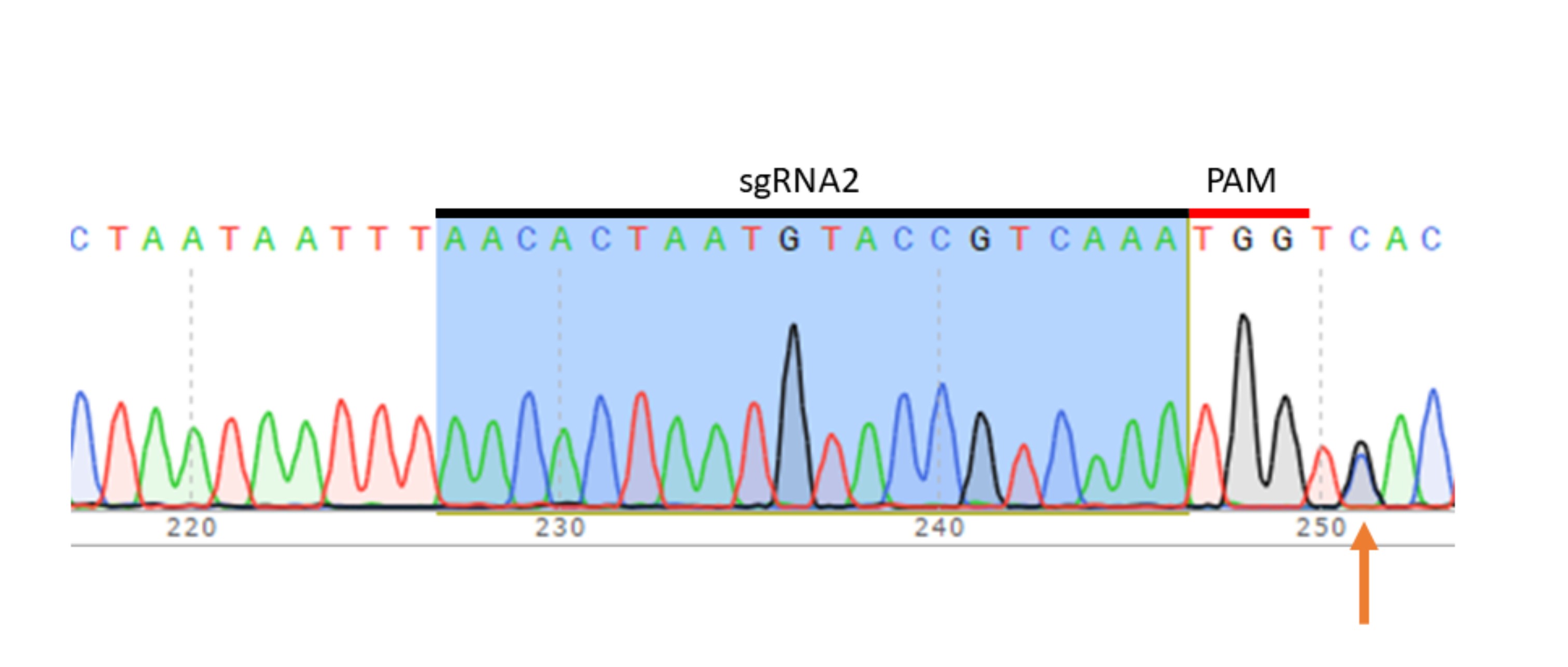

Supplement: Supplementary Figure 1 — Chromatogram of PPO1 in wildtype DRH195. Orange arrow indicates presence of mixed template. [file Image1.jpeg]

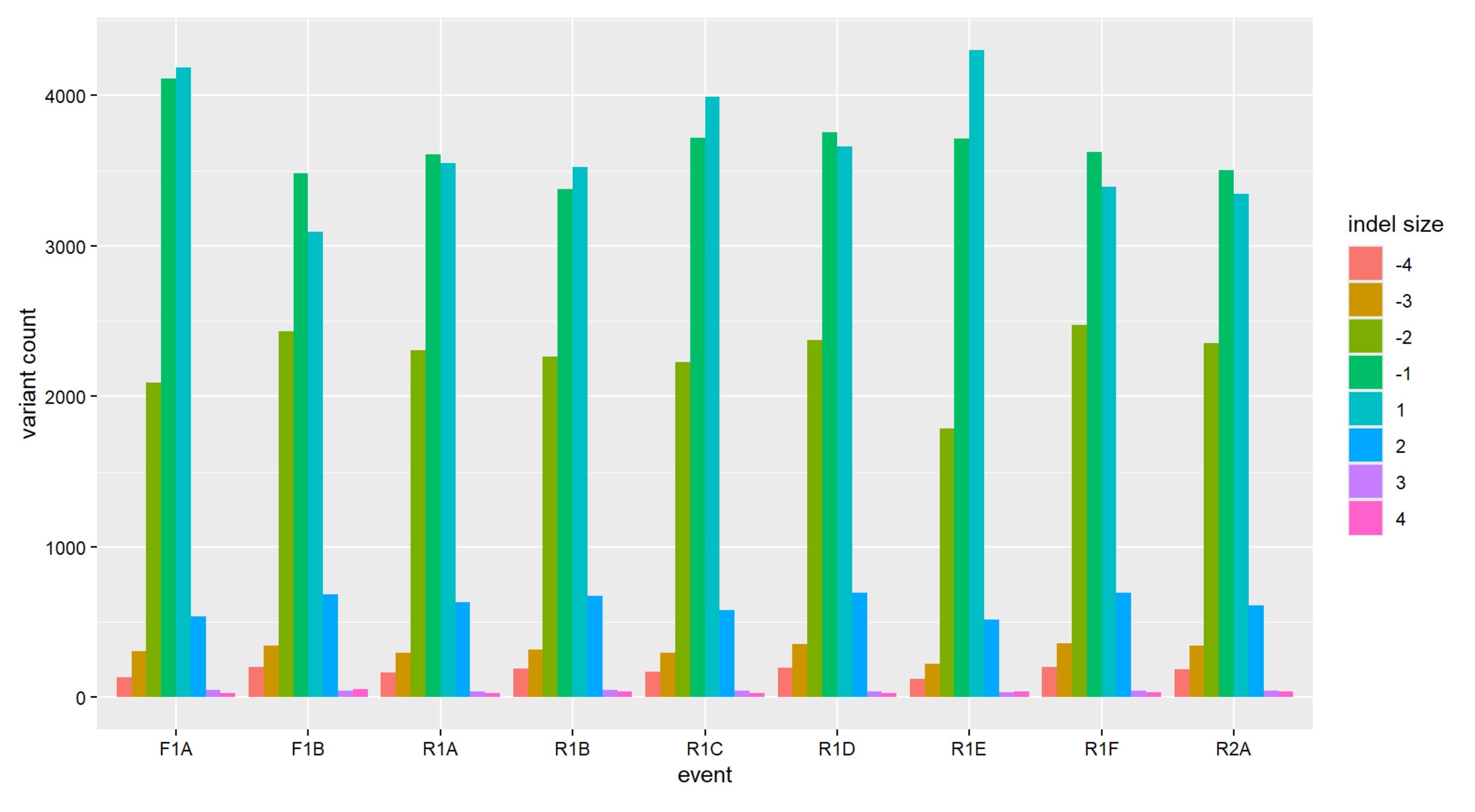

Supplement: Supplementary Figure 2 — Summary of indel lengths from -4 to 4 basepairs for all camelina events. [file Image2.png]
